# Impact of clinical and molecular features on efficacy and outcome of patients with non-small cell lung cancer receiving second-line osimertinib

**DOI:** 10.1186/s12885-022-09683-1

**Published:** 2022-05-28

**Authors:** Ying Jin, Chen Lin, Xun Shi, Qiong He, Junrong Yan, Xinmin Yu, Ming Chen

**Affiliations:** 1 The Second Affliated Hospital of Soochow University, Suzhou, 215004 Jiangsu China; 2 The Cancer Hospital of the University of Chinese Academy of Sciences (Zhejiang Cancer Hospital), Hangzhou, 310022 Zhejiang China; 3 Institute of Basic Medicine and Cancer (IBMC), Chinese Academy of Sciences, Hangzhou, 310022 Zhejiang China; 4 Zhejiang Key Laboratory of Radiation Oncology, Hangzhou, 310022 Zhejiang China; 5 Medical Department, Nanjing Geneseeq Technology Inc, Nanjing, 210000 China; 6 Department of Radiation Oncology, Sun Yat-Sen University Cancer Center, Guangzhou, 510060 Guangdong China; 7 State Key Laboratory of Oncology in South China, Collaborative Innovation Center for Cancer Medicine, Sun Yat-Sen University, Guangzhou, 510060 Guangdong China

**Keywords:** Clinical and molecular features, Efficacy and outcome, EGFR T790M mutation, Non-small-cell lung cancer, Osimertinib

## Abstract

**Background:**

Although with the impressive efficacy, several patients showed intrinsic resistance or an unsatisfactory response to Osimertinib. We aim to explore the impact of clinical and molecular features on efficacy and outcome of patients with *EGFR* T790M-mutation non-small cell lung cancer (NSCLC) receiving second-line Osimertinib.

**Methods:**

Patients with *EGFR* T790M-mutant NSCLC who had acquired resistance to the first-generation EGFR TKI and then received Osimertinib as second-line treatment were included. Patients’ demographic and clinical information, as well as molecular data were extracted from electronic medical records. The impact of clinical and molecular features on treatment response and patients’ outcome were assessed.

**Results:**

Among the 99 patients, 60 patients were tissue/pleural effusion T790M positive and 69 patients were plasma positive with a median PFS of 12.1 m and 9.9 m (*P* = 0.25), respectively. In addition, median PFS were similar between patients of plasma T790M + and patients of plasma T790M- (*P* = 0.94). The Pearson correlation test showed no significant relationship between plasma T790M abundance and PFS (*r* = 0.074, *P* = 0.546). In subgroup analyses, PFS was significantly improved in elder patients (*P* = 0.009) and patients with longer PFS to the first-generation EGFR TKI (*P* = 0.0008), while smokers tended to have worse PFS compared with non-smokers (*P* = 0.064). *PARP1* mutant-type patients had a worse PFS compared with wild-type group (*P* = 0.0003). Patients with *MYC* amplification also had a worse PFS than *MYC* wild-type patients (*P* = 0.016). A significant PFS shrinkage was observed in TMB-High group as 6.77 m, compared with 19.10 m in TMB-Low group. The multivariate Cox analysis revealed that years ≥ 65 was an independent positive feature for PFS, while *PARP1* mutation and TMB-H were negative features for PFS.

**Conclusion:**

In conclusion, our findings in this study demonstrated that clinical and molecular features can be served as predictive biomarkers to stratify patients with EGFR T790M-mutant NSCLC receiving second-line Osimertinib.

**Supplementary information:**

The online version contains supplementary material available at 10.1186/s12885-022-09683-1.

## Background

The discovery of epidermal growth factor receptor (*EGFR*) driver mutation has led to a dramatic paradigm shift in the therapeutic strategies for advanced non-small-cell lung cancer (NSCLC). However, despite high initial responses to first-/second-generation EGFR tyrosine kinase inhibitors (TKIs) and a median progression-free survival (PFS) of 10 to 14 months, resistance eventually develops in the majority of patients [1–6]. The most common mechanism of resistance, which accounts for ~ 60% of cases, is the acquired mutation T790M, namely threonine-to-methionine substitution at codon 790 [7].

Osimertinib is an oral third-generation EGFR TKI that selectively inhibits both *EGFR*-sensitive and T790M-resistant mutations, which has been approved as a standard of care after disease progression against first- generation (1^st^-G)/second-generation(2^nd^-G) EGFR TKIs in patients with mutant T790M, based on the results of the AURA series studies [8–10]. In a tissue-based assay, the objective response rate (ORR) ranged from 62 to 71%, and the median PFS ranged from 9.6 to 12.3 months.

In clinical practice, tumor re-biopsies for detecting T790M mutation in advanced NSCLC are not always feasible for patients with late-stage disease. Plasma cfDNA mutation testing has unique advantages by reducing risks of complications and overcoming tumor heterogeneity [11]. However, whether the efficacy of Osimertinib in patients with T790M mutation identified by the cfDNA is equal to those identified by the tissue samples is not fully addressed. In addition, the correlation between mutant allele frequency (MAF) and drug responsiveness is an unmet needs.

Although the impressive efficacy of osimertinib, 30–40% of patients have limited response [8, 10, 12]. Targeted therapy typically induces an incomplete tumor response due to the Intratumor heterogeneity. Recent studies have found co-occurring genomic alterations were common in *EGFR*-mutated lung cancers, especially in advanced-stage cancers [13]; however, the biological significance of these co-occurring events and their correlation with clinical features were largely unknown. Although T790M mutation is a defined mechanism of 1^st^ and 2^nd^-G TKI resistance, co-occurring somatic alterations associated with the innate resistance and subgroups would show distinct clinical outcomes have not been systematically investigated. Elucidating the correlation between molecular characteristics and treatment response, as well as identifying subgroups with poor efficacy, are essential in enhancing therapeutic responses.

In this study, we have investigated the clinical and molecular features predicting the efficacy and outcome of patients with *EGFR* T790M-mutant NSCLC receiving second-line Osimertinib retrospectively.

## Patients and methods

### Patient enrollment

This study was performed in line with the principles of the Declaration of Helsinki. All methods were performed in accordance with the relevant guidelines and regulations. Patients with *EGFR* T790M-mutant NSCLC who had acquired resistance to the 1^st^-G EGFR TKI and then received Osimertinib as second-line treatment from June 1, 2015 to Dec 31, 2019 at the Cancer Hospital of the University of Chinese Academy of Sciences (Zhejiang Cancer Hospital) were included. Targeted NGS of post-treatment tumor samples had been performed before Osimertinib treatment for these patients. The NGS platform was Nanjing Geneseeq Technology Inc. (Nanjing, Jiangsu, China). Patients’ demographic and clinical information, as well as molecular data were extracted from electronic medical records. The histological classification was based on the World Health Organization Criteria (2015 version) [14].

### DNA extraction and quantification

6–10 of 6–10 μm FFPE sections from tumor samples were used for genomic DNA extraction with QIAamp DNA FFPE Tissue Kit (QIAGEN) following the manufacturer’s instructions. Plasma was extracted from 8–10 ml peripheral blood in EDTA-coated tubes within 2 h of blood withdrawing. Circulating cell free DNA (cfDNA) from plasma and pleural effusions (PE) supernatants was extracted using the Circulating Nucleic Acid Kit (QIAGEN). Genomic DNA from the white blood cells were extracted using the DNeasy Blood & Tissue Kit (QIAGEN) and used as the normal control to remove germline variations.. The DNA quality was assessed by a Nanodrop2000 (Thermo Fisher Scientific) and the quantity was measured by dsDNA HS Assay Kit on Qubit 3.0 (Life Technologies).

### Library preparation and targeted NGS

Extracted tumor genomic DNA was fragmented into 300 ~ 350 bp using Covaris M220 instrument (Covaris). Sequencing libraries were prepared with KAPA Hyper Prep kit (KAPA Biosystems) with optimized protocols. In brief, cfDNA or sheared tissue DNA were experienced with end-repairing, A-tailing, adapter ligation and size selection using Agencourt AMPure XP beads (Beckman Coulter). Libraries were then subjected to PCR amplification and purification before targeted enrichment.

Indexed DNA libraries were pooled up to 2 µg together with Human cot-1 DNA (Life Technologies) and xGen Universal blocking oligos (Integrated DNA Technologies) as blocking reagents. Customized xGen lockdown probes panel (Integrated DNA Technologies) were used to selectively enrich for 425 or 139 predefined genes. The enriched libraries were sequenced on Hiseq 4000 NGS platforms (Illumina) with 2 × 150 bp pair-end reads to coverage depths of at least 100x, 600x, 5000 × for blood, FFPE, and cfDNA, respectively.

### Bioinformatics analyses

Sequencing data were demultiplexed by bcl2fastq (v2.19), analyzed by Trimmomatic (Bolger et.al., 2014) to remove low-quality (quality < 15) or N bases. Then the data were aligned to the reference human genome (build hg19) with the Burrows–Wheeler Aligner (bwa-mem) [15] and further processed using the Picard suite (http://picard.sourceforge.net/) and the Genome Analysis Toolkit (GATK). Common SNPs were removed using dbSNP and the 1000 Genome data sets. Germline mutations were filtered out by comparing to the whole blood controls. A mutation was called when the MAF cutoff was ≥ 0.5% for tissue samples, 0.1% for liquid biopsy samples, and a minimum of three unique mutant reads on different strands with good quality scores and manually inspected in Integrative Genomics Viewer Software (IGV, Broad Institute). Gene fusions were identified by FACTERA [16] and copy number variations (CNVs) were analyzed with ADTEx [17]. The log2 ratio cut-off for copy number gain was defined as 2.0 for tissue samples and 1.6 for liquid biopsy samples. A log2 ratio cut-off of 0.6 was used for copy number loss detection in all sample types. Tumor mutation burden (TMB) was defined as the number of somatic, coding, base substitution, and indel mutations per megabase of genome examined, and was calculated as previously described [18]. Briefly, all base substitutions, including non-synonymous and synonymous alterations, and indels in the coding region of targeted genes were considered with the exception of known hotspot mutations in oncogenic driver genes and truncations in tumor suppressors. Synonymous mutations were counted in order to reduce sampling noise and known driver mutations were excluded as they are over-represented in the Panel.

### Assessment of efficacy and follow-up

All patients were radiologically evaluable according to Response Evaluation Criteria in Solid Tumors (RECIST) version 1.1 [19] through spiral Computed tomography (CT) or magnetic resonance imaging (MRI) scans. PFS was defined as the time from the first medication to the first objective progression of disease or the date of death from any causes. Outpatient or telephonic follow-up was adopted, and the last follow-up time of this study was on April 21, 2020.

### Statistical analyses

Quantitative data were displayed as median (range) or number of patients (percentage). Comparisons of proportion between two groups were done using the Fisher’s exact test. The Pearson correlation coefficient was used to determine the association of PFS with the allele frequency (AF) of *EGFR* T790M detected in plasma cfDNA. Wilcoxon rank-sum test was performed to compare the TMB between different sub-groups of PFS of 1^st^-G EGFR TKI (PFS1). Differences in AF of *EGFR* T790M between groups of best overall response (BOR) of osimertinib were measured by the Kruskal–Wallis test. For survival analyses, Kaplan–Meier curves were compared using the log-rank test, and hazard ratios (HRs) and the 95% confidence intervals (CIs) were calculated by Cox proportional hazards model. Univariate and multivariate analyses were performed to study the association between different variables and PFS. A two-sided *P* value of less than 0.05 was considered significant for all tests unless indicated otherwise. All statistical analyses were done in R (v.3.6.0).

## Results

### Patient cohort and clinical characteristics

A total of 99 EGFR T790M + patients who received second-line Osimertinib were included in the study. The demographics and clinical characteristics of 99 NSCLC patients are presented in Table 1, including age, gender, smoking status, pathological type, EGFR mutant subtype, metastatic sites, BOR and median PFS1. There were 62 men and 37 women with a median age of 61 years (range, 36–80 years). Sixty-two (62.6%) patients originally harbored EGFR exon19 deletion and 37 (37.4%) harbored L858R mutation. The objective response rate for 1^st^-G EGFR TKI was 75.8%. The median PFS was 12.43 months (range: 3.2–51.8 months).

**Table 1:** Baseline clinical characteristics of the study population

Characteristics	Population (%)
**Median Age, years (range)**	61 (36–80)
≥ 65	30 (30.3%)
< 65	69 (69.7%)
**Gender**	
Male	37 (37.4%)
Female	62 (62.6%)
**Smoking status**	
Ever	27 (27.3%)
Never	72 (72.7%)
**Histology**	
ADC	98 (99.0%)
SCC	1 (1.0%)
***EGFR* Mutant**	
19del	62 (62.6%)
L858R	37 (37.4%)
**CNS metastasis**	
Yes	16 (16.2%)
No	83 (83.8%)
**Liver metastasis**	
Yes	11 (11.1%)
No	88 (88.9%)
**Bone metastasis**	
Yes	47 (47.5%)
No	52 (52.5%)
**Median PFS of 1**^**st**^**-generation EGFR-TKI, months (95%CI)**	12.43 (3.2–51.8)
≥ 12 months	53 (53.5%)
< 12 months	46 (46.5%)
**BOR of 1**^**st**^**-generation EGFR-TKI**	
SD	24 (24.2%)
PR	75 (75.8%)
**Pleural effusion**	
** With**	45 (45.5%)
** Without**	54 (54.5%)

*TKI* Tyrosine kinase inhibitor, *BOR* Best of response, *SD* Stable disease, PR Partial response, ADC Adenocarcinoma, *SCC* Squamous cell carcinoma, *CNS* Central nervous system, *CI* Confidence interval, *PFS* Progression-free survival

Of the 99 patients, targeted sequencing of 425 cancer genes was performed in 77 patients, while 139 gene panel was performed in 22 patients on account of lower cost before Osimertinib treatment. For NGS data analyses, after excluding samples from three patients without negative control samples, samples from 96 patients were included into analyses. Among the 76 patients whose samples were performed by targeted sequencing of 425 cancer genes, 48 had both paired tissue/pleural effusion and plasma samples, 4 patients only had tissue/pleural effusion samples and 4 patients only had plasma samples. Among the 20 patients whose samples were performed by targeted sequencing of 139 cancer genes, 12 had both paired tissue/pleural effusion and plasma samples, 2 patients only had tissue samples and 6 patients only had plasma samples (Fig. S1).

### Genomic landscape of tissue/pleural effusion and plasma samples

**Fig. 1:**
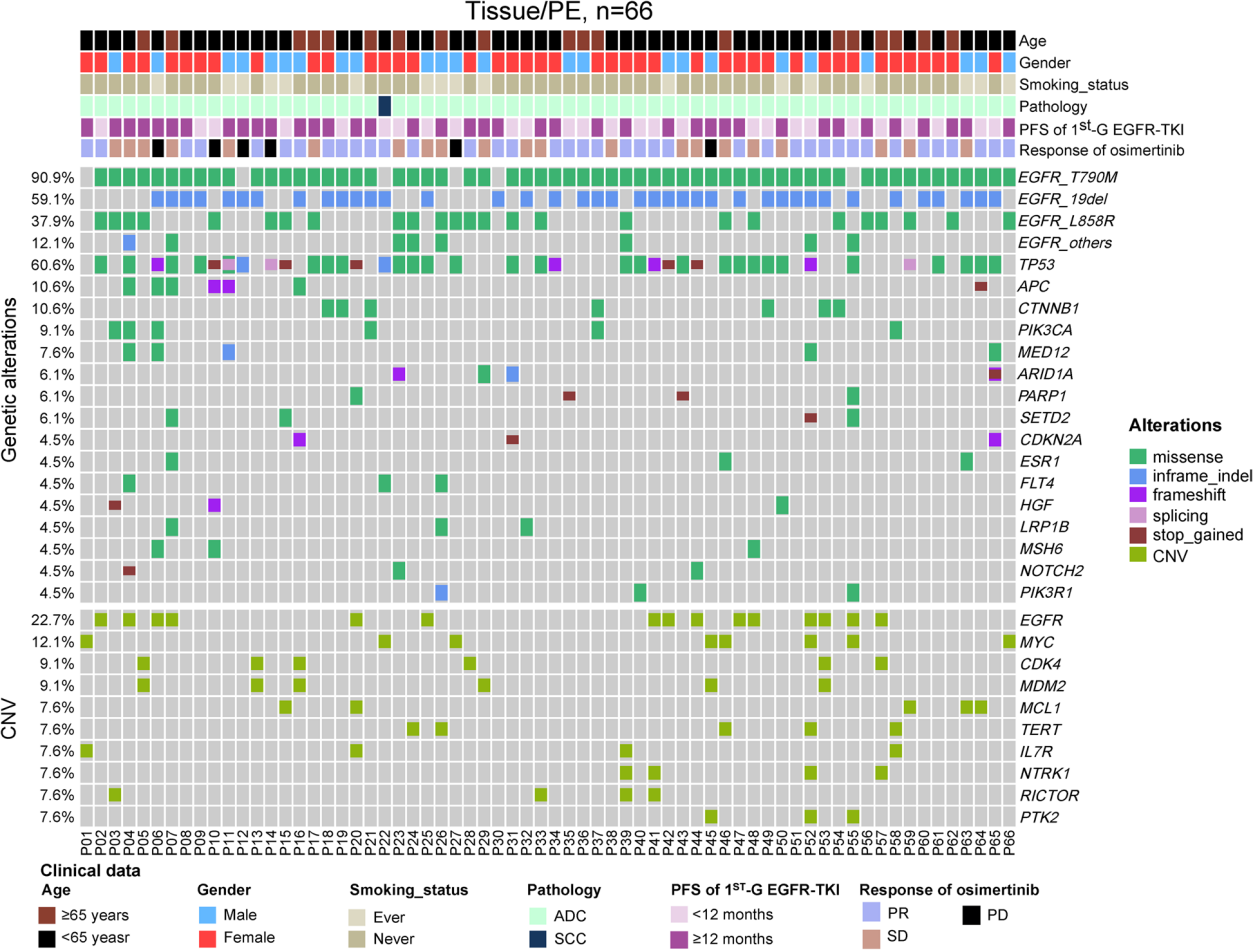
Genomic landscape of 66 patients who underwent tissue or pleural effusion sequencing. Baseline mutations and SCNAs of the most frequently changed genes were detected by next-generation sequencing of 66 patients with *EGFR*-mutant NSCLC prior to Osimertinib treatment

The genomic landscape of 66 patients with tissue (*n* = 43) or plural effusion (*n* = 23) performed by NGS are shown in Fig. 1. Among them, sixty (90.0%) patients were identified with *EGFR* T790M mutation, with 22/23 (95.7%) pleural effusion were T790M+, and 38/42 (88.4%) tissue were T790M+. Of the 66 patients, 59.1% were identified with Exon19 Del, 37.9% with L858R mutation and 12.1% with other sites mutations of *EGFR*. Widespread genetic alterations were co-occurring with *EGFR* mutations, with *TP53* (60.6%) being the most frequently commutated genes. Several genes that have been reported in NSCLC [13, 20, 21] were found to be co-occurred with *EGFR*, including *APC* (10.6%), *CTNNB1* (10.6%), *PIK3CA* (9.1%), *MED12* (7.6%), *ARID1A* (6.1%), *PARP1* (6.1%), *SETD2* (6.1%), *CDKN2A* (4.5%). 22.7% of patients had *EGFR* amplification, which is the most frequent SCNA in our cohort, followed by *MYC* amplification (12.1%), *CDK4* amplification (9.1%) and *MDM2* amplification (9.1%). In addition, the genomic landscape of 90 patients with plasma performed by NGS are depicted in Figure S2.

### The efficacy of Osimertinib based on tissue or plasma T790M mutation

**Fig. 2:**
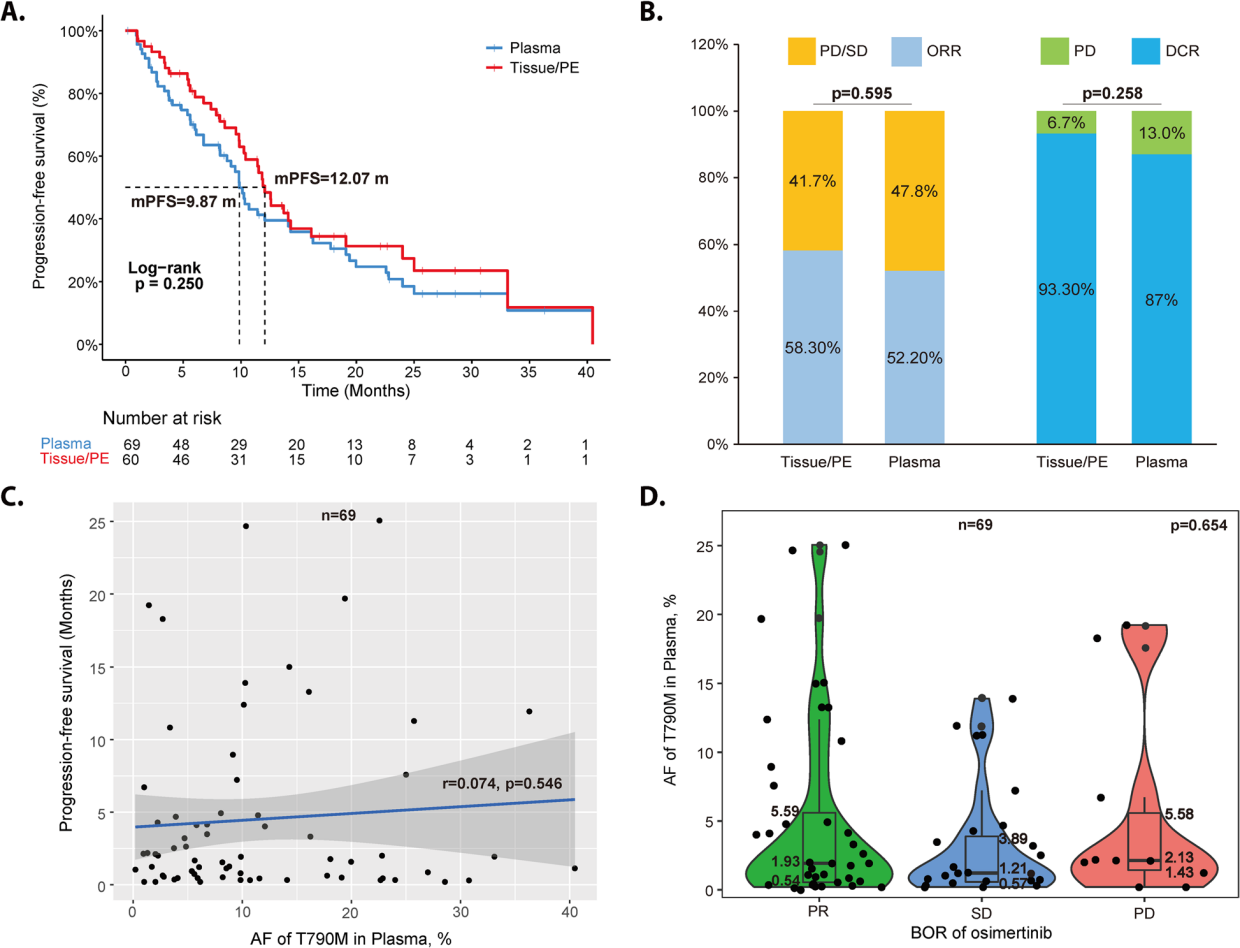
The efficacy of Osimertinib based on tissue or plasma T790M mutation. **A** Comparison of PFS between patients with tissue/pleural effusion T790M mutation and those with plasma T790M mutation. **B** Comparison of ORR and DCR between patients with tissue/pleural effusion T790M mutation and those with plasma T790M mutation. **C** Correlation analysis of plasma T790M AF and PFS. **D** Comparison of plasma T790M AF among three groups according to the BOR to Osimertinib

Among the 99 patients who were detected with T790M mutation by tissue or plasma, the ORR of Osimertinib was 53.5% and the median PFS was 10.3 m (Fig. S3). Separately, 60 patients were tissue/pleural effusion T790M positive and 69 patients were plasma positive with a median PFS of 12.1 m and 9.9 m (*P* = 0.25), respectively (Fig. 2A). The ORR and DCR in the tissue/pleural effusion T790M + group were 58.3% and 98.3%, compared with 52.2% and 87% in the plasma T790M + group (Fig. 2B). In addition, median PFS were similar between patients of plasma T790M + and patients of plasma T790M- (*P* = 0.94; Fig. S4). We also explored the relationship between plasma T790M abundance and the efficacy of Osimertnib. The median T790M abundance was 1.9%, ranging from 0.2%-25.1% and the pearson correlation test showed no significant relationship between plasma AF and PFS (*r* = 0.074,*P* = 0.546) (Fig. 2C). When patients were divided into three groups according to the BOR to Osimertinib, the median plasma T790M mutation-allele frequency (MAF) was similar as 1.93% vs 1.21% vs 2.13% among PR, SD and PD groups (*P* = 0.654) (Fig. 2D).

### Subgroup analyses of PFS to Osimertinib

**Fig. 3:**
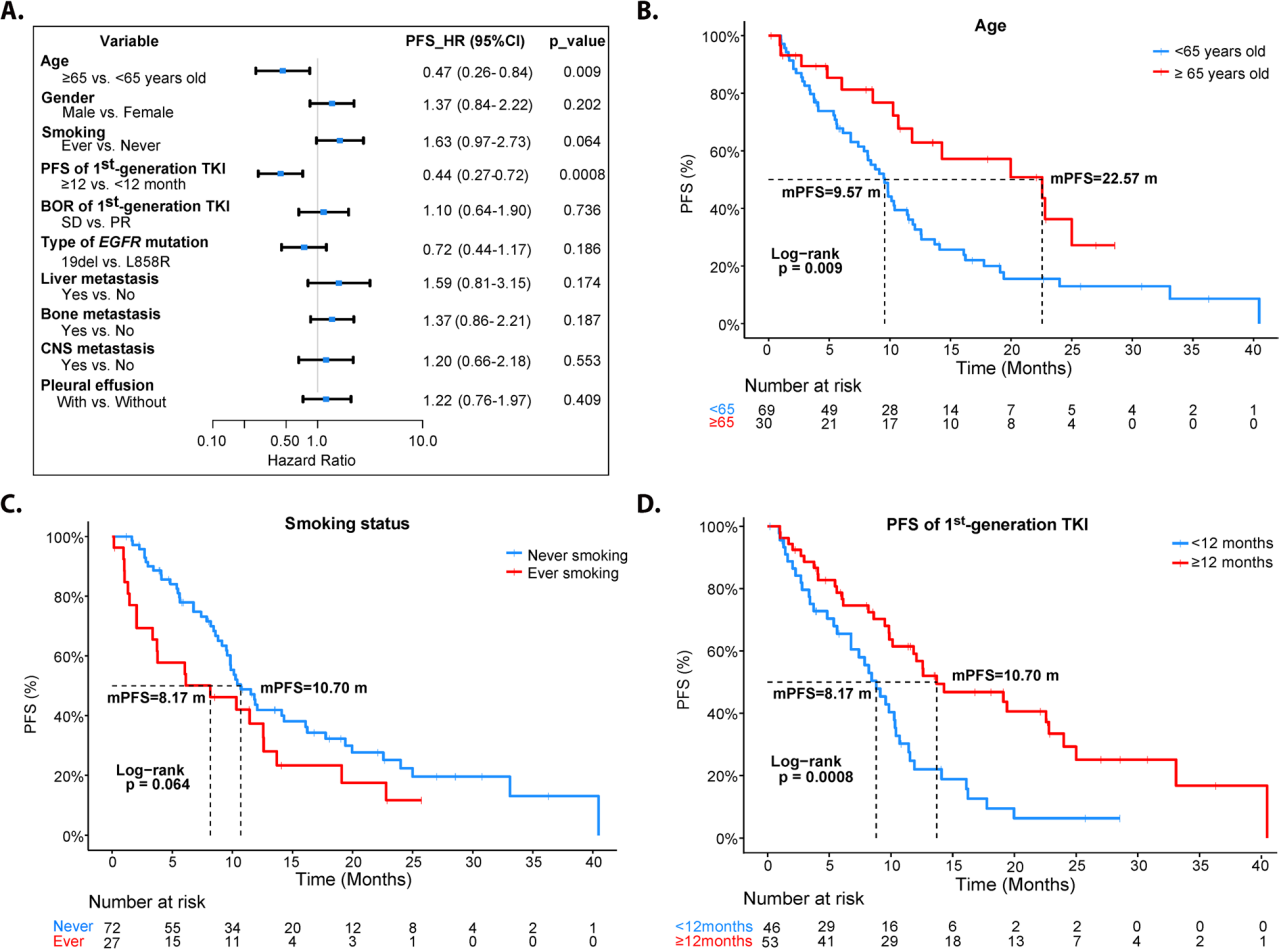
Subgroup analyses of PFS to Osimertinib. **A** Forest plot of hazard ratio (HR) and *p* value for ultivariate analyses of clinical subgroup. **B** Comparison of PFS between elder patients (≥ 65 years)and younger patients (< 65 years). **C** Comparison of PFS between patients of smoker and patients of non-smoker. **D** Comparison of PFS between patients with longer PFS1 (≥ 12 months) and patients with shorter PFS1 (< 12 months)

In subgroup analyses, PFS was significantly improved in elder patients (HR = 0.47, 95%CI 0.26–0.84, Log-rank *p* = 0.009) and patients with longer PFS to the 1^st^ -G EGFR TKI (HR = 0.44, 95%CI 0.27–0.72, Log-rank *p* = 0.0008), while smokers tended to have worse PFS compared with non-smokers (HR = 1.63, 95%CI 0.97–2.73, Log-rank *p* = 0.064). No significant difference of PFS was detected in other subgroups like gender, BOR to the 1^st^ -G EGFR TKI, *EGFR* mutation subtype and metastatic sites (Fig. 3A-D; Fig. S5; Fig. S6).

### Baseline genetic alterations and TMB affected the clinical response to Osimertinib

**Fig. 4:**
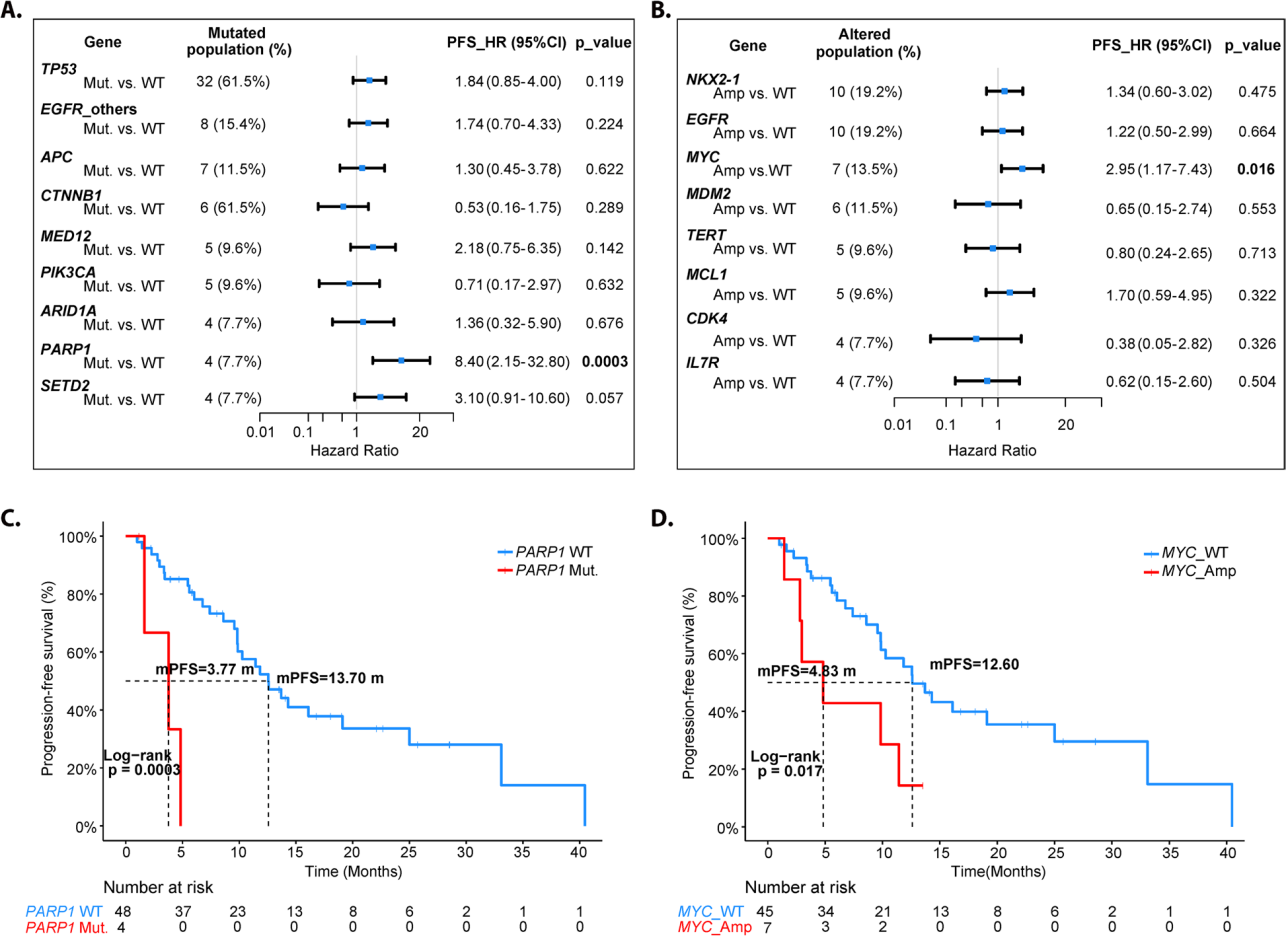
Baseline genetic alterations affected the clinical response to Osimertinib. **A** Forest plot of hazard ratio (HR) and p value for ultivariate analyses of somatic mutations identified in more than 4 patients. **B** Forest plot of hazard ratio (HR) and p value for ultivariate analyses of SCNAs identified in more than 4 patients. **C** Comparison of PFS between *PARP1* mutant-type patients and *PARP1* wild-type patients. **D** Comparison of PFS between Patients with *MYC* amplification and *MYC* wild-type patients

In order to explore the effects of co-occurring genetic alterations on the clinical response to Osimertinib, somatic mutations and SCNAs in more than 4 patients were identified and linked to PFS (Fig. 4A-B). The results demonstrated that *PARP1* mutant-type patients had a worse PFS compared with wild-type group (3.77 m vs. 13.70 m, HR = 8.4, Log-rank *p* = 0.0003). Patients with *MYC* amplification also had a worse PFS than *MYC* wild-type patients (4.83 m vs 12.60 m, HR = 2.95, Log-rank *p* = 0.016) (Fig. 4C-D).

**Fig. 5:**
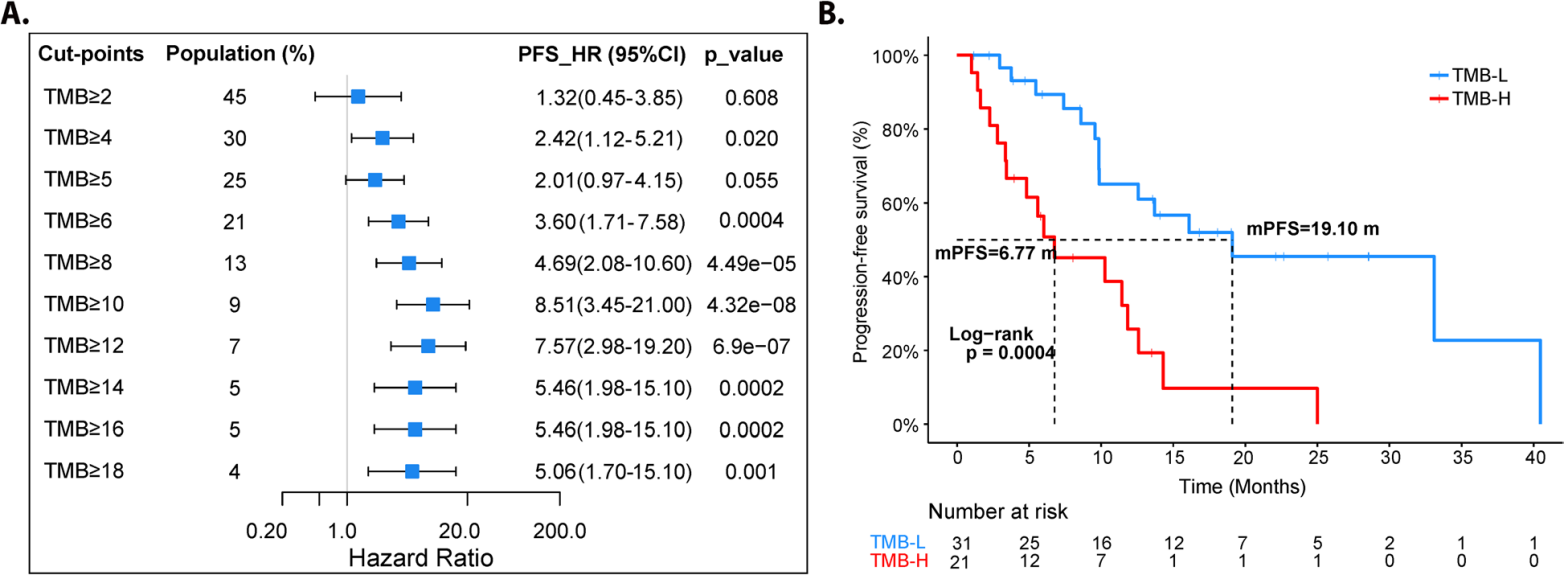
TMB was associated with the clinical response to Osimertinib. **A** Forest plot of hazard ratio (HR) and p value for ultivariate analyses of different TMB-H cut-off value. **B** Comparison of PFS between patients of TMB-H (≥ 6 mutations/MB) and patients of TMB-L (< 6 mutations/MB)

Of the 66 patients with tissue/plural effusion, 55 patients were performed by NGS of 425 panel. We then elucidate the correlation between TMB and PFS by consecutively setting TMB cut-points. When TMB high was defined as TMB ≥ 6, a significant PFS shrinkage was observed in TMB-High group as 6.77 m, compared with 19.10 m in TMB-Low group. The trend remained along with higher threshold value of TMB-High (Fig. 5).

### Multivariate Cox analyses of PFS

**Fig. 6:**
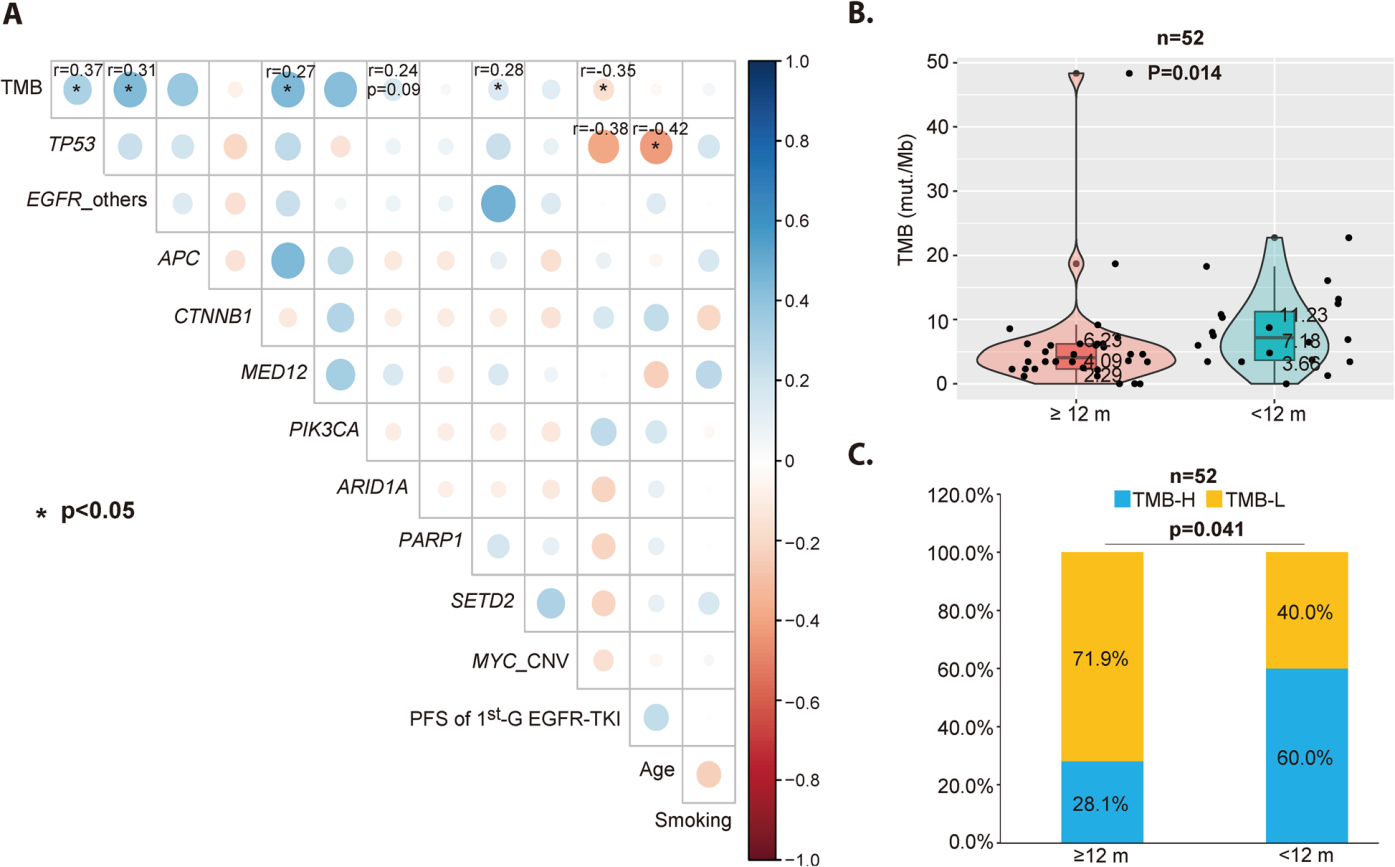
Correlation analysis between TMB and multiple variables. **A** Correlation analyses between TMB and multiple variables. **B** Comparison of TMB value between patients with longer PFS1 (≥ 12 months) and shorter PFS1 (< 12 months). **C** Comparison of the percentage of TMB-H between patients with longer PFS1 (≥ 12 months) and shorter PFS1 (< 12 months)

The multivariate Cox analysis was conducted incorporating clinical and genetic features that were found significant in univariate analysis, which was displayed in Table 2. The results revealed that years ≥ 65 was an independent positive feature for PFS, while *PARP1* mutation and TMB-H were negative features for PFS. Interestingly, PFS1 was no longer a prognostic factor in multivariate Cox analysis. We speculated whether it is possible that PFS1 influences PFS of Osimertinib through the number of coincidental mutations, therefore, correlation analysis was conducted between TMB and multiple variables. As a result, PFS1 did have a moderate negative correlation with TMB (Fig. 6A). By T-test, we found there were significantly fewer patients with TMB-H in patients with longer PFS1 (*P* = 0.041) (Fig. 6B).

**Table 2:** Univariate and multivariate Cox analysis (*n* = 52)

Variable	PFS (months)	Univariate analysis		Multivariate analysis	
		**HR (95% CI)**	***P* value**	**HR (95% CI)**	***P* value**
**Age (years)** ≥ 65 vs. < 65	22.57 vs. 9.57	0.47 (0.26–0.84)	0.009	0.18 (0.06–0.51)	**0.001**
**PFS of 1**^**st**^**-G EGFR-TKI** ≥ 12 vs. < 12 months	10.70 vs. 8.17	0.44 (0.27–0.72)	0.0008	0.67 (0.28–1.62)	0.379
***TP53*** Mut. vs. WT	9.87 vs. 14.30	1.62 (0.85–3.10)	0.119	0.55 (0.21–1.41)	0.213
***PARP1*** Mut. vs. WT	3.77 vs. 13.70	8.40 (2.15–32.80)	0.0003	11.39 (2.45–52.82)	**0.002**
***MYC*** Amp vs. WT	4.83 vs. 12.60	2.95 (1.17–7.43)	0.016	1.19 (0.42–3.40)	0.744
**TMB (Mut./Mb)** TMB-H vs. TMB-L	6.77 vs. 19.10	3.6 (1.71–7.58)	0.0004	6.65 (2.59–17.04)	**8.00E-05**

*HR* Hazard ratio, *WT* Wild-type, *Amp* Amplification, *Mut* Mutation, *PFS* Progression-free survival

## Discussion

Osimertinib is a third-generation EGFR TKI, which has been approved for the treatment of patients with EGFR T790M-mutant NSCLC whose disease progress after 1^st^- or 2^nd^-G EGFR TKIs. Although with the impressive efficacy, several patients showed intrinsic resistance or an unsatisfactory response to Osimertinib, similar to that observed with other TKIs [22]. Here we explored potential biomarkers based on clinical and genetic features to predict the efficacy and outcome of *EGFR* T790M-mutant NSCLC patients treated with second-line Osimertinib.

In the AURA3 trial, the ORR and median PFS of patients treated with Osimertinib was 71% and 10.1 m, better than with the chemotherapy group (ORR:31%, PFS:4.4 m) [10]. In our real-world study, the ORR of Osimertinib as the second-line therapy in patients with acquired EGFR T790M mutation was 53.5% and the median PFS was 10.3 m. A meta-analysis including 3086 advanced NSCLC patients from 11 studies reported that the aggregate efficacy parameters for advanced NSCLC harboring T790M mutations after earlier-generation EGFR-TKI therapy are as follows: ORR 58%, DCR 80%, PFS 10.58 m [23].

In this study, we demonstrated that the ORR and outcome were similar between patients with tissue/pleural effusion T790M + and those with cfDNA T790M + . In addition, median PFS were similar between patients of plasma T790M + and patients of plasma T790M-. We also explored the relationship between plasma T790M abundance and the efficacy of Osimertnib and the results showed no significant relationship between plasma MAF and PFS. Another research also reported that ORR and median PFS were similar in patients with T790M-positive plasma (ORR, 63%; PFS, 9.7 months) or T790M-positive tumor (ORR, 62%; PFS, 9.7 months) results [24]. These results suggest that, cfDNA T790M assay is a validated surrogate to identify patients who will have benefit from Osimertinib that are similar to patients with T790M mutation tested by tissue samples. On the other hand, a negative T790M result by liquid biopsy should be validated by tissue testing, to determine the T790M status, if possible.

The main point of this study is to identify clinical and molecular features predicting outcome in NSCLC patients receiving Osimertinib. Firstly, we conducted the ultivariate analyses of PFS in different subgroups based on clinical features. The results revealed that PFS was significantly improved in elder patients and patients with longer PFS to the 1^st^ -G EGFR TKI, while smokers tended to have worse PFS compared with non-smokers. In a PRISMA-compliant systematic review and meta-analysis including 47 studies evaluating the efficacy and safety of Osimertinib in treating NSCLC, risk factors associated with survival outcome were analyzed. The results also showed that patients who have a smoking history may have a higher risk of progression than never-smoker patients [25]. The worse survival outcome and the lower response rate to the EGFR TKI in smokers with activating EGFR mutations might be attributed to several reasons. High number of SNVs and structural mutation might be associated with smoking, which may decrease the efficacy of EGFR TKI [26, 27]. In addition, downstream activation of AKT, ERK pathway and Src signaling pathways via nicotine exposure might induce tumor growth [28, 29]. In several other research exploring how clinical characteristics affected the efficacy of Osimertinib therapy, elder patients exhibited higher response rates and better PFS [30, 31]. One of the reasonable explaination is the higher prevalence of uncommon EGFR mutations in younger patients, that are known to be less sensitive to EGFR TKIs. In addition, younger patients were more likely to have brain metastasis at baseline and to be heavily pretreated [32, 33].

In this study, we observed extensive accompanying genomic alterations that co-occurred with T790M mutations after the 1^st^-G EGFR TKI resistance, both in the tissue samples and in the plasma samples. Although T790M mutation is a major resistance mechanism for 1^st^- and 2^nd^-G EGFR TKIs, growing evidence revealed that other co-acquired alterations may cooperate with T790M. In our previous study about the resistant mechanism of 1^st^-G EGFR TKIs, we also demonstrated that T790M was accompanied by other acquired oncogenic alterations, especially gene mutations [34]. Recent studies have found co-occurring genomic alterations were common in EGFR-mutated lung cancers. These accompanying alterations can affect the response of the tumors to EGFR TKIs, which may serve as predictive biomarkers to stratify patients and explore the novel treatment paradigm [13].

We then explored the effects of co-occurring genetic alterations on the clinical response to Osimertinib. The results demonstrated that *PARP1* mutation was associated with worse PFS. PARP1 is the most abundant enzyme mediating post-translational polyADP-ribosylation, which is involved in DNA repair, transcriptional control, genomic stability, and cell death [35]. A research using cell experiments and GEO dataset analysis confirmed that PARP1 was expressed at higher levels in TKI-resistant cells than in TKI-sensitive cells. PARP1-mediated autophagy was a key pathway for TKI resistance in NSCLC cells that participated in the resistance to TKIs. Olaparib may serve as a novel method to overcome the resistance to TKIs [36]. Another study revealed that acquired resistance of EGFR-mutated lung cancer to TKI treatment promoted PARP inhibitor sensitivity [37]. Despite the fact that these results still need to be validated in larger cohorts, they are indeed with potential implications that cytotoxic anticancer drug or PARP inhibitors combining with current standard TKI therapy may improve the prognosis of this subgroup of patients.

Furthermore, in our study, TMB high (TMB ≥ 6) was identified to be an independent negative prognostic factor for PFS. TMB is an emerging positively predictive marker of immune checkpoint blockade response in several malignancies including lung cancer, which is hypothesized to be associated with the increased tumor-specific neoantigen burden [38]. In contrast, its utility in targeted therapy is still not addressed. It is hypothesized that the elevated TMB may correlate with additional mutations which represent potential pathways for resistance to targeted therapies. Analogous results were found by previous research demonstrating that TMB was negatively associated with clinical outcomes in metastatic patients with *EGFR*-mutant lung cancer treated with first- or second- generation EGFR TKIs [13, 39].

## Conclusions

In this study, several clinical and molecular characteristics were identified to be associated with treatment outcome in patients with EGFR T790M-mutant NSCLC receiving second-line Osimertinib. The multivariate Cox analysis revealed that years ≥ 65 was an independent positive feature, while *PARP1* mutation and TMB-H were negative features for PFS. These findings demonstrated that clinical and molecular features can be served as predictive biomarkers to stratify patients treated with second-line Osimertinib.

## Supplementary information

**Additional file 1:** **Fig. S1.** Flow chart of patients and samples exclusion.

**Additional file 2:** **Fig. S2.** Effect of Osimertinib of the included cohort (*n* = 99).

**Additional file 3:** **Fig. S3.** The genomic landscape of 90 patients with plasma performed by NGS.

**Additional file 4:** **Fig. S4.** Comparison of PFS between patients of plasma T790M+ and patients of plasma T790M-.

**Additional file 5:** **Fig. S5.** Subgroup analyses of PFS to Osimertinib. **A** Comparison of PFS between patients with Ex19 Del and patients with Ex 21L858R. **B** Comparison of PFS between patients with PR and patients with SD for 1^st^ -G EGFR TKI. **C** Comparison of PFS between patients with and without CNS metastasis. **D** Comparison of PFS between patients with and without liver metastasis. **E** Comparison of PFS between patients with and without bone metastasis. **F** Comparison of PFS between patients with and without pleural effusion.

**Additional file 6:** **Fig. S6.** Forest plot of hazard ratio (HR) and p value for ultivariate analyses of different PFS1 cut-off value.

## Data Availability

The datasets analysed during the current study are available from the corresponding author on reasonable request.

## Abbreviations

EGFR: Epidermal growth factor receptor
NSCLC: Non-small-cell lung cancer
TKIs: Tyrosine kinase inhibitors
1^st^-G: First- generation
2^nd^-G: Second-generation
PFS: Progression-free survival
ORR: Objective response rate
NGS: Next-generation sequencing
cfDNA: Cell free DNA
PE: Pleural effusions
MAF: Mutant allele frequency
CNVs: Copy number variations
TMB: Tumor mutation burden
RECIST: Response Evaluation Criteria in Solid Tumors
CT: Computed tomography
MRI: Magnetic resonance imaging
AF: Allele frequency
PFS1: PFS of 1^st^-G EGFR TKI
BOR: Best overall response
HRs: Hazard ratios
Cis: Confidence intervals
