# Supplementary figures and images for: Impact of clinical and molecular features on efficacy and outcome of patients with non-small cell lung cancer receiving second-line osimertinib
Source: BMC Cancer. 2022 May 28;22:586. doi: 10.1186/s12885-022-09683-1 (PMC9145492; doi:10.1186/s12885-022-09683-1)

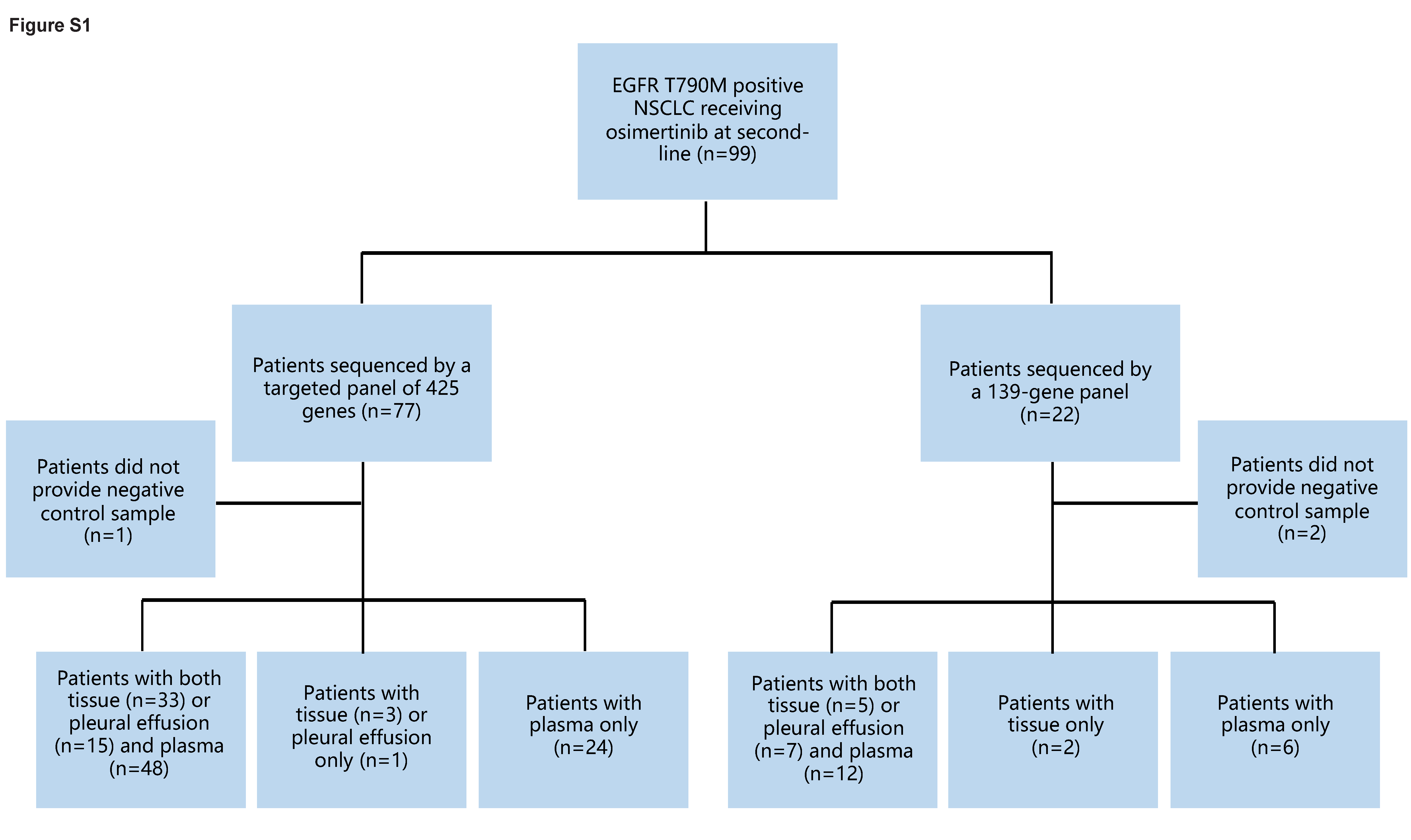

Supplement: Supplementary file 1 — Additional file 1: Fig. S1. Flow chart of patients and samples exclusion. [file 12885_2022_9683_MOESM1_ESM.tif]

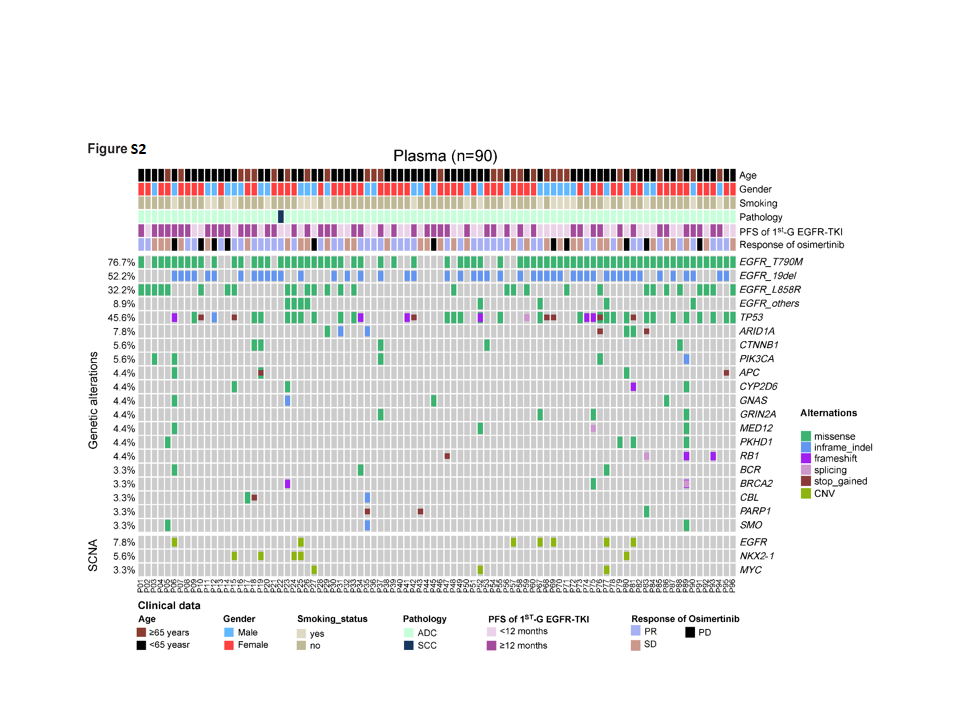

Supplement: Supplementary file 2 — Additional file 2: Fig. S2. Effect of Osimertinib of the included cohort (n = 99). [file 12885_2022_9683_MOESM2_ESM.tif]

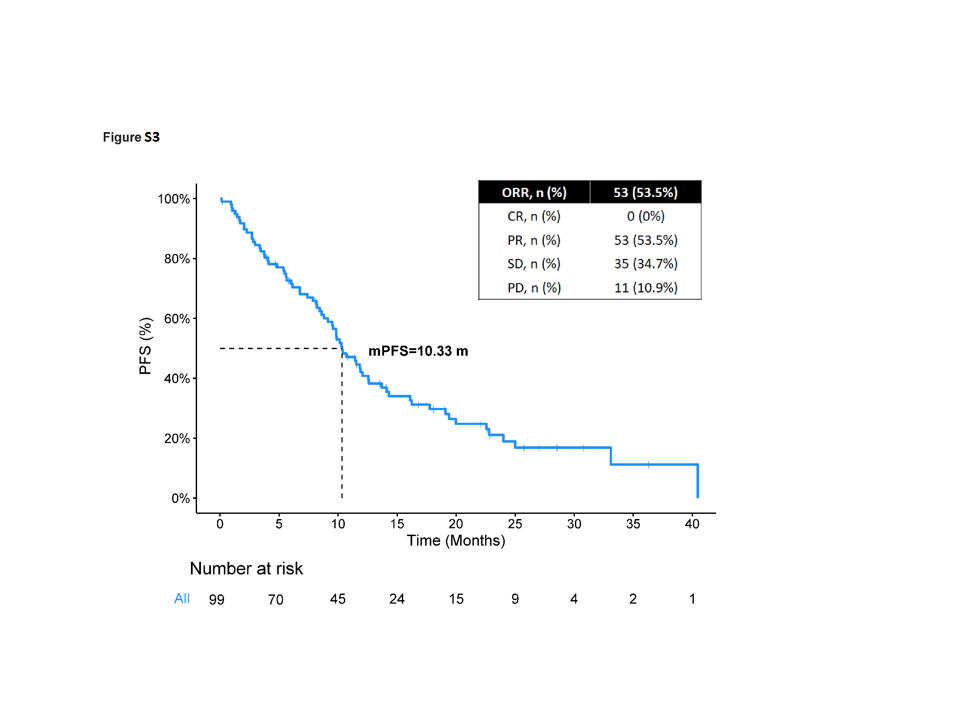

Supplement: Supplementary file 3 — Additional file 3: Fig. S3. The genomic landscape of 90 patients with plasma performed by NGS. [file 12885_2022_9683_MOESM3_ESM.tif]

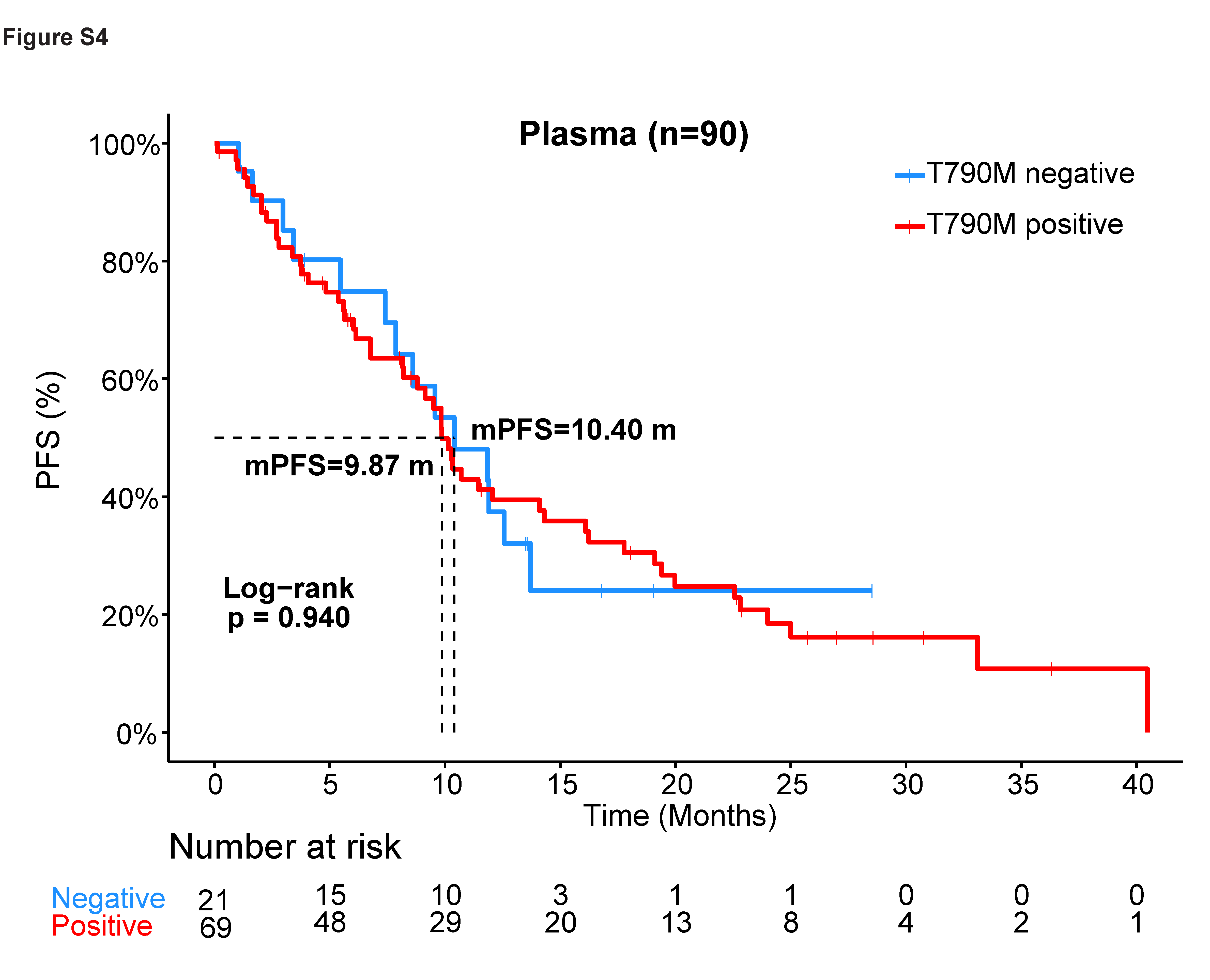

Supplement: Supplementary file 4 — Additional file 4: Fig. S4. Comparison of PFS between patients of plasma T790M+ and patients of plasma T790M-. [file 12885_2022_9683_MOESM4_ESM.tif]

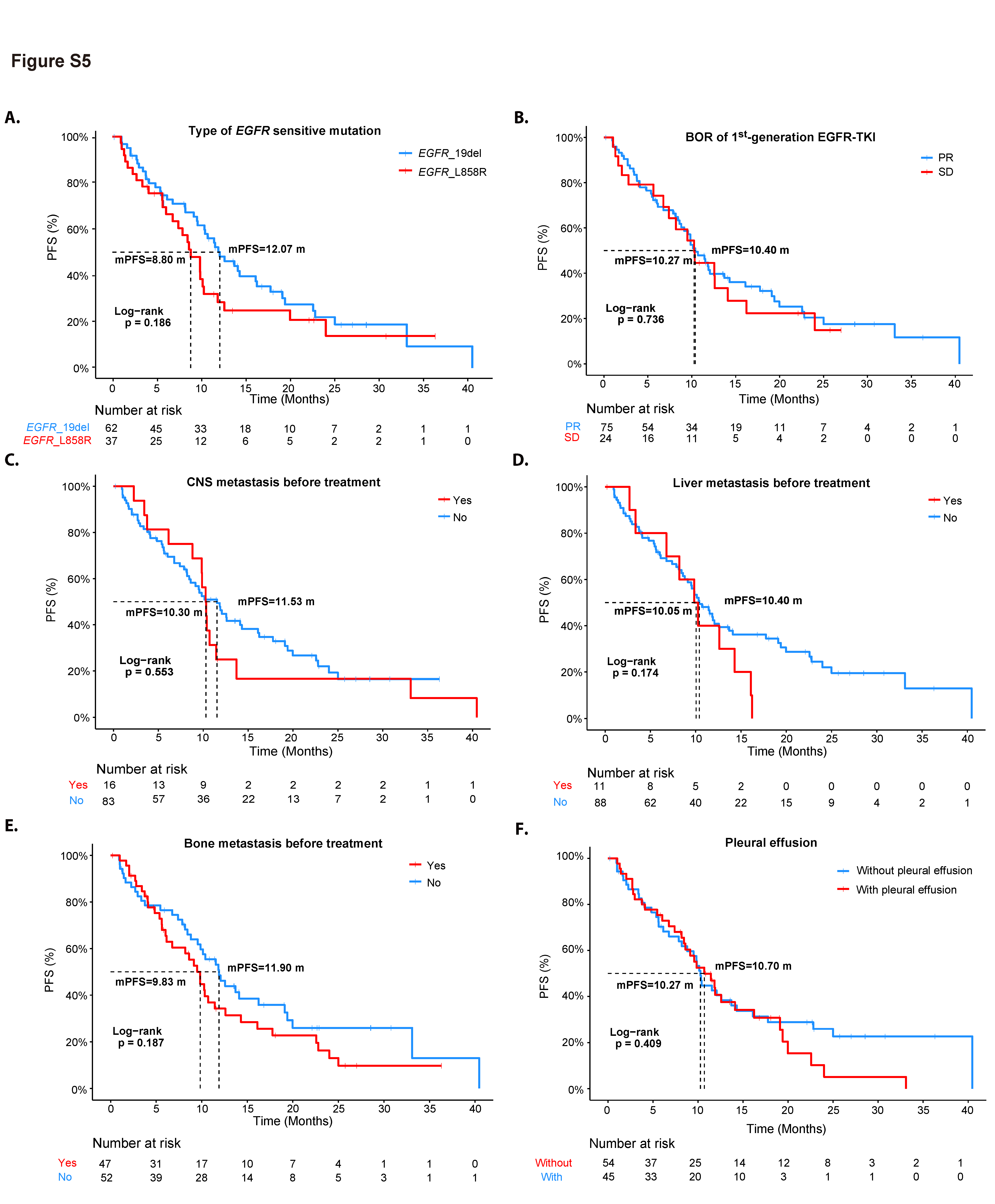

Supplement: Supplementary file 5 — Additional file 5: Fig. S5. Subgroup analyses of PFS to Osimertinib. A Comparison of PFS between patients with Ex19 Del and patients with Ex 21L858R. B Comparison of PFS between patients with PR and patients with SD for 1st -G EGFR TKI. C Comparison of PFS between patients with and without CNS metastasis. D Comparison of PFS between patients with and without liver metastasis. E Comparison of PFS between patients with and without bone metastasis. F Comparison of PFS between patients with and without pleural effusion. [file 12885_2022_9683_MOESM5_ESM.tif]

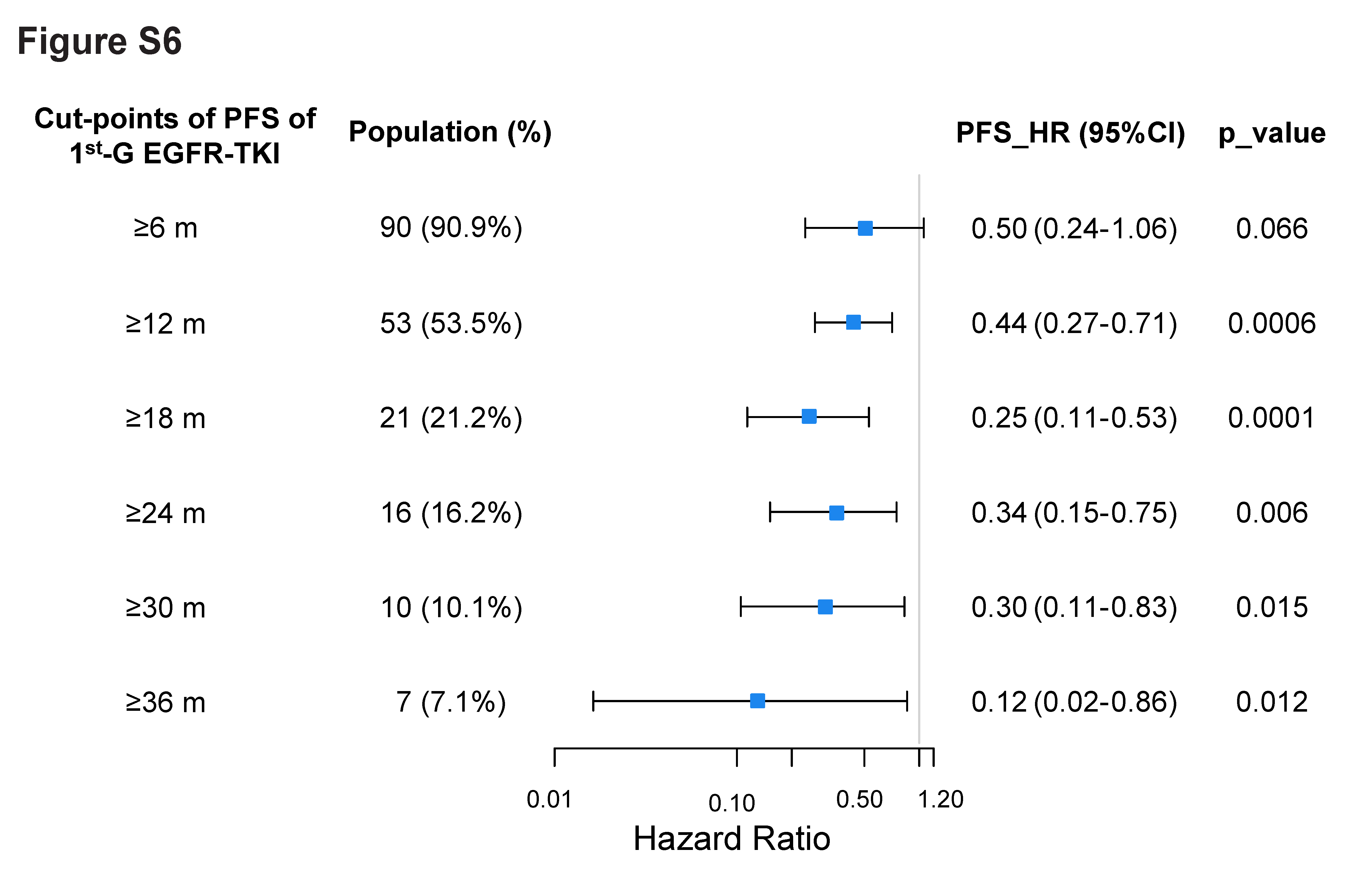

Supplement: Supplementary file 6 — Additional file 6: Fig. S6. Forest plot of hazard ratio (HR) and p value for ultivariate analyses of different PFS1 cut-off value. [file 12885_2022_9683_MOESM6_ESM.tif]
